# Perceived discrimination and subjective well-being of left-behind children: social support and psychological resilience as mediators

**DOI:** 10.3389/fpsyg.2025.1660514

**Published:** 2025-12-15

**Authors:** Weifeng Xue, Yidan Ma, Yin Xu

**Affiliations:** 1 Institute of Education Science, Leshan Normal University, Leshan, China; 2 Sichuan Center for Rural Education Development Research, Leshan Normal University, Leshan, China; 3 School of Public Administration, Sichuan University, Chengdu, China

**Keywords:** left-behind children, perceived discrimination, psychological resilience, social support, subjective well-being

## Abstract

**Introduction:**

Chinese left-behind children (LBC) report perceiving significant discrimination, a chronic stressor linked to adverse mental health outcomes. However, the mechanisms underlying this association remain unclear. Guided by the stress process model, which suggests that exposure to stress can increase the risk of poorer mental health through the depletion of psychosocial resources, this study examined whether the association between perceived discrimination and subjective well-being was partially explained by social support and psychological resilience.

**Methods:**

A cross-sectional study was conducted in Sichuan Province, southwest China. Questionnaires on Perceived Discrimination, Social Support Rating Scale, Resilience Scale for Chinese Adolescents, and Subjective Happiness Scale were completed by 719 LBC (aged 10–15 years) from primary and junior high school.

**Results:**

We found that higher perceived discrimination was significantly associated with lower subjective well-being. This association was partially explained by social support and psychological resilience directly, as well as by a path involving lower social support leading to reduced psychological resilience.

**Discussion:**

These findings contribute to understanding the mechanism through which discrimination may influence LBC’s subjective well-being. This highlights the need for multi-level interventions that aim to enhance individual resilience, strengthen social support networks, and address the broader issue of discrimination.

## 1 Introduction

China’s rapid urbanization has attracted millions of rural migrant workers to urban centers in pursuit of economic advancement. However, most of these workers have to leave their children in their hometowns because they cannot afford to raise them in urban settings (Wang et al., 2022). This has resulted in 41.77 million children known as “left-behind children” (LBC) (United Nations Children’s Fund, 2023). LBC are rural children left at home for extended periods, typically over 6 months, when one or both of their parents migrate to urban areas for work (Zhao et al., 2020). The psychological well-being of LBC has become an increasing concern and attracted considerable public attention in recent years. Research has demonstrated that parental migration is associated with increased risk of poorer mental health among LBC (Fellmeth et al., 2018; Hu et al., 2022; Lin et al., 2025). A well-documented manifestation of this effect is their markedly lower subjective well-being compared to other children (Li and Liu, 2013; Ye et al., 2020). This pattern has been consistently identified in studies conducted across Europe, Africa, America, and Asia (Graham and Jordan, 2011; Jordan and Graham, 2012; Mazzucato et al., 2015; Bălţătescu et al., 2023). As a multidimensional construct encompassing both individuals’ cognitive and affective assessments of their lives (Diener, 2000), subjective well-being serves as a critical measure of mental health (Diener et al., 1999). Therefore, identifying the factors and mechanisms that shape LBC’s subjective well-being is essential for developing effective interventions.

Perceived discrimination, defined as the perception of being treated differently or unfairly owing to membership or classification within a certain group (Pascoe and Richman, 2009), functions as a chronic stressor (Thoits, 2010) and has been identified as a significant threat to mental health (Pascoe and Richman, 2009; Schmitt et al., 2014). Within the context of LBC in China, children are labeled as “left-behind,” leading them to perceive both individual-level and group-level discrimination. The absence of parental care exposes these children to stigmatization, such as being ridiculed as “unwanted children,” which fosters individual-level discrimination. Additionally, media coverage highlighting behavioral issues among some LBC has reinforced negative public stereotypes, further branding them as “problem children,” and contributing to group-level discrimination (Zhao et al., 2016, 2020; Hu et al., 2022). Consistent with previous studies (Pascoe and Richman, 2009; Schmitt et al., 2014; Adedeji et al., 2025), higher levels of perceived discrimination were associated with lower levels of subjective well-being among LBC (Shen et al., 2009; Zhang et al., 2020). In addition, this association was not moderated by sex (Pascoe and Richman, 2009; Zhao et al., 2016; Emmer et al., 2024). The stress process model offers a theoretical framework for understanding the association between perceived discrimination and subjective well-being (Pearlin et al., 1981; Pearlin, 1989; Wheaton, 2010; Turner, 2013). Specifically, this model suggests that exposure to stress may lead to increased risk of poorer mental health either directly through the activation of the stress process or indirectly through the depletion of psychosocial resources (Pearlin, 1989; Wheaton, 1994; Turner, 2013). Importantly, empirical evidence indicates that although there are sex differences in stress exposures, these pathways tend to operate similarly for both sexes, with limited support for sex as a moderating factor (Pascoe and Richman, 2009; Turner, 2013; Anderson et al., 2022). Within this model, stress generally encompasses life events and chronic strains, such as chronic illness and perceived discrimination (Pearlin, 2010). Psychosocial resources generally include social resources (e.g., social support) and personal resources (e.g., mattering and self-esteem). Although the stress process model has received substantial empirical support (Cairney et al., 2013; Aneshensel and Mitchell, 2014), its application among vulnerable populations, such as LBC, remains limited. Given the central role of psychosocial resources in this framework, it is essential to examine specific mechanisms–particularly social support–through which discrimination may affect the subjective well-being of LBC.

Social support is defined as spiritual and material support (e.g., love, care, respect) obtained from social relationships like family, colleagues, groups, organizations, and communities (Cobb, 1976). Previous research has consistently found that greater social support is associated with higher levels of subjective well-being (Yalcin, 2015; Siedlecki et al., 2014) and is especially vital for LBC lacking parental care. According to the stress process model, perceived discrimination can undermine social support, reducing the protective resources available to cope with stress and thereby increasing the risk of lower levels of subjective well-being. In addition, the social support deterioration deterrence model also provides insight into the potential mediating pathway between perceived discrimination and mental health outcomes via social support. This model posits that a stressor such as perceived discrimination (Vines et al., 2017) may inhibit the protective effects of social support on an individual’s mental health (Kaniasty and Norris, 1993; Norris and Kaniasty, 1996). Indeed, empirical studies across socio-cultural groups (Prelow et al., 2006; Jia and Liu, 2017), including left-behind children (Fu et al., 2024), demonstrate that perceived discrimination significantly diminishes social support, which in turn leads to poorer mental health. Therefore, social support may partially explain the association between perceived discrimination and subjective well-being among LBC.

In addition to eroding social resources, exposure to stress may be also associated with lower levels of subjective well-being through eroding personal resources (Pearlin, 1989; Wheaton, 1994). Psychological resilience is defined as the adaptability and ability to remain healthy despite experiencing stress and adversity (Ungar, 2012). While resilience is a protective factor again poorer mental health (Fritz et al., 2018; Ungar and Theron, 2020), including among LBC (Han et al., 2021), it can be eroded by chronic stressors, as suggested by the stress process model. Symbolic interactionist theory (Brownfield and Thompson, 2005) provides a specific mechanism for this effect: perceived discrimination (a form of negative social feedback) promotes the internalization of stigma (Fernández et al., 2015), which in turn lowers self-evaluation (Szalacha et al., 2003), thereby resulting in reduced psychological resilience (Olsson et al., 2003). Indeed, studies on LBC support this pathway, showing that higher levels of perceived discrimination were associated with reduced resilience, which is in turn correlated with poorer school adjustment and more severe emotional and behavioral problems among LBC (Han and Long, 2020; Lv et al., 2021). Therefore, psychological resilience may partially explain the relationship between perceived discrimination and subjective well-being among LBC.

Social support and psychological resilience can each independently partially explain the association between perceived discrimination and subjective well-being, and social support may also influence psychological resilience, resulting in a potential chain mediation effect. Specifically, the association between perceived discrimination and subjective well-being may be partially explained by a path involving the depletion of social resources (social support) leading to the erosion of personal resources (psychological resilience). The strong link between social support and psychological resilience is well-established in the literature. According to the resilience framework (Kumpfer, 2002), stable and diverse social ties can provide external support resources for individuals to adapt to adversity, and subsequently produce positive outcomes (Noh and Park, 2022). Extensive evidence suggests that social support is closely related to psychological resilience and, particularly, resilience development in children (Olsson et al., 2003; Zimmerman, 2013; Masten, 2018). Research has also shown that higher levels of social support are strongly correlated with higher levels of resilience among LBC (Huang et al., 2022; Nguyen et al., 2023). Recent studies indicate that resilience may partially explain the relationship between social support and mental health (i.e., subjective well-being and satisfaction with life) (Fan and Lu, 2020; Yıldırım and Tanrıverdi, 2021). Furthermore, existing research proposes a serial mediation model in which the effects of stigma on mental health outcomes among people living with HIV are sequentially explained by a path through lower levels of social support leading to reduced psychological resilience (Yan et al., 2019; Yuan et al., 2024), highlighting their potential role in explaining the relationship between discrimination and subjective well-being. For LBC, we hypothesize a similar serial pathway. Based on the above-mentioned information, we hypothesized that the association between perceived discrimination and subjective well-being would be partially explained by a path through lower levels of social support leading to reduced psychological resilience.

In summary, guided by the stress process model, the current study aims to further our understanding of the mechanisms underlying the relationship between perceived discrimination and subjective well-being among LBC by exploring the role of social support and psychological resilience using a serial mediation model. Based on previous studies and theories, we hypothesized that: (H1) perceived discrimination is negatively correlated with subjective well-being among LBC; (H2) social support would partially explain the association between perceived discrimination and subjective well-being; (H3) psychological resilience would partially explain the relationship between perceived discrimination and subjective well-being; and (H4) the association between perceived discrimination and subjective well-being would also be partially explained by the path through lower levels of social support leading to reduced psychological resilience. Finally, consistent with evidence that the pathway from exposure to stress to subjective well-being via the depletion of psychosocial resources operates similarly across sex (Pascoe and Richman, 2009; Turner, 2013; Anderson et al., 2022), we also hypothesized that sex would not moderate any of the indirect effects of social support or psychological resilience in the association between perceived discrimination and subjective well-being (H5).

## 2 Methods

### 2.1 Participants

A cross-sectional study was conducted in Sichuan Province, southwest China. Sichuan has the second largest number of migrant workers. Over six million laborers from Sichuan migrated to other provinces for long-term employment between 2010 and 2020 (Xiao et al., 2022), resulting in the province having the largest population of LBC in China (Tang et al., 2020). Consequently, it serves as a valuable data source for studying mental health issues among this vulnerable population. In selecting the sample, this study took both representativeness and economic feasibility into account. Accordingly, whole-group sampling was used to recruit a cohort of 1010 students, including 499 Grade 5 and 6 pupils from two elementary schools, as well as 511 Grade 7 and 8 pupils from two junior high schools located in rural regions. These schools were selected for being typical rural schools with high rates of parental migration.

Those who refused to participate or were absent from school were excluded from the study. Among the respondents, 63 were excluded due to questionnaires with missing values exceeding 5% or containing obviously false responses, which resulted in 947 valid questionnaires (valid rate of 93.76%). Then, LBC were identified through their answers to “one or both parents away from home for work more than 6 months.” Finally, 719 LBC were included in our analysis. Among them, 232 (32.27%) were single-parent LBC and 487 (67.73%) were two-parent LBC; 344 (47.84%) were boys and 375 (52.16%) were girls. Their age ranged from 10 to 15 years (*M* = 12.45, SD = 0.98). The descriptive statistics of social demographic variables are presented in Table 1.

**TABLE 1:** Descriptive statistics of social demographic variables (*N* = 719).

Characteristics	Frequency (*N*)	Percentage (%)
**Sex**		
Female	375	52.16%
Male	344	47.84%
Age	Mean = 12.45	S.D. = 0.98
**Situation of parents**		
Father works outside	180	25.03%
Mother works outside	52	7.23%
Both work outside	487	67.73%
**Grade**		
Grade 5	159	22.11%
Grade 6	192	26.70%
Grade 7	300	41.72%
Grade 8	68	9.46%
**Family socioeconomic status**		
High	27	3.76%
Upper middle	127	17.66%
Medium	487	67.73%
Lower middle	64	8.90%
Low	14	1.95%
**Educational level (father)**		
Illiterate	3	0.42%
Primary school	139	19.33%
Junior high school	310	43.12%
Senior high school	159	22.11%
College degree or higher	33	4.59%
Unknown	75	10.43%
**Educational level (mother)**		
Illiterate	6	0.83%
Primary school	150	20.86%
Junior high school	308	42.84%
Senior high school	136	18.92%
College degree or above	32	4.45%
Unknown	87	12.10%

According to power simulation (Thoemmes et al., 2010), the sample size surpasses the required minimal sample size for detecting a small chain indirect effect of social support and psychological resilience on the association between perceived discrimination and subjective well-being at the 5% significance level and 90% power (the path from perceived discrimination to subjective well-being was set to have a *R*^2^ of 0.02, which is considered to be a small effect; each remaining path, including the path from perceived discrimination to social support, path from social support to psychological resilience, and the path from psychological resilience to subjective well-being, was set to have a *R*^2^ of 0.13, which is considered to be a medium effect (Cohen, 1988).

### 2.2 Procedure

Prior to the survey, informed written consent was obtained from the parents, or guardians on behalf of the participating minors (<16 years). Furthermore, verbal consent was obtained from each participant. They then completed the paper-and-pencil survey in class, after which each participant received a ballpoint pen as a gift. Participants were told there were no right or wrong answers and they could withdraw from the study at any time. All participants were explicitly assured that their responses would be confidential and anonymous, and data would be stored securely.

The survey was administrated by six undergraduate psychology students who had received standardized training prior to data collection. This training covered the research objectives, ethical principles of working with minors, and a standardized protocol for survey administration. To ensure procedural consistency and minimize potential bias, the protocol included a verbatim script for introducing the study and specific guidelines on how to answer participants’ questions neutrally (e.g., re-reading the item or response options without interpretation). For Grade 5 and 6 pupils, the trained administrators read each item aloud from the script and provided standardized explanations for any difficult terms, while pupils marked their responses on the paper survey. For Grade 7 and 8 pupils, the administrators first read the overall instructions aloud using the script, after which the pupils completed their questionnaires independently. It took 20–30 min to complete the questionnaires.

The Survey and Behavioral Research Ethics Committee at the first author’s university approved the research procedures. Permission was granted to conduct the study by the local government and by school administrators or principals.

### 2.3 Measurement

#### 2.3.1 Sociodemographic questionnaire

A self-assessment questionnaire on sociodemographic characteristics was conducted to collect basic information on the participants, including age, sex (male or female), grade (Grade 5, Grade 6, Grade 7, or Grade 8), parental educational level (illiterate, primary school, junior high school, senior high school, college degree or higher, unknown), situation of parents (father works outside, mother works outside, or both parents work outside), and subjective family socioeconomic status (high, upper middle, medium, lower middle, low).

#### 2.3.2 Perceived discrimination

The Perceived Discrimination Questionnaire was a six-item Chinese scale assessing perceived discrimination in Chinese LBC (Shen et al., 2009). This scale has acceptable reliability and validity (Zhao et al., 2016, 2020), Cronbach’s α was 0.86 in the current study. An example item is “In general, I feel that students whose family situations are like mine have been treated unfairly.” Responses range from 1 = strongly disagree to 5 = strongly agree. The total score of the six items was used in the analysis. Higher scores indicate a greater perception of discrimination.

#### 2.3.3 Social support

The 10-item Chinese Social Support Rating Scale (Xiao, 1994), revised to reflect the situation of LBC (Liu et al., 2007), was used to measure social support received by LBC. This scale has acceptable reliability and validity (Liu et al., 2007; Xing et al., 2017). Cronbach’s α was 0.79 in the current study. This scale comprises three dimensions: subjective support (3 items, e.g., “How about your relationship with neighbors?”), objective support (4 items, e.g., “What are your sources of comfort and concern when you are in an emergency?”), and utilization of social support (3 items, e.g., “How do you seek for help when you have trouble?”). The total score of the ten items was used in the analysis. Overall, higher scores indicated higher levels of personal social support.

#### 2.3.4 Psychological resilience

The Resilience Scale for Chinese Adolescents was a validated measure to rate the psychological resilience of LBC (Hu and Gan, 2008; Tian et al., 2019). Cronbach’s α was 0.84 in the current study. It comprises 27 items across five dimensions–goal concentration, emotion regulation, positive perception, family support, and interpersonal assistance–and includes two second-order factors–personal strength and support strength. As psychological resilience mainly refers to an individual’s capability or trait, only the former subscale is used (Song and Wang, 2017). It comprises 15 items that include statements such as “I have a clear goal in my life,” answered on a five-point Likert scale, ranging from 1 = strongly disagree to 5 = strongly agree. The total score of the 15 items was used in the analysis. Higher scores indicate better resilience.

#### 2.3.5 Subjective well-being

Subjective well-being was measured using the Subjective Happiness Scale (Lyubomirsky and Lepper, 1999). Subjective happiness and subjective well-being are synonymous here (Diener, 2000). This scale has been successfully used with Chinese LBC, demonstrating good validity and reliability (Dai and Chu, 2018; Zhao et al., 2019). Cronbach’s α was 0.79 in the current study. The scale includes four general items about happiness, such as “I am very happy,” rated on a seven-point Likert-type scale ranging from 1 = very unhappy to 7 = very happy. The total score of the four items was used in the analysis. Higher scores indicate higher levels of subjective well-being.

### 2.4 Data analysis

Discriminant validity and multicollinearity were examined using SmartPLS 4.1.1.4, while all other analyses were performed in Mplus 8.3. Pearson correlations were used to test the relationships among perceived discrimination, subjective well-being, social support, and psychological resilience. The assumption for linear association was not violated based on scatter plots. Robust likelihood estimation (MLR) was used to handle potential violation of normality.

Subsequently, in order to test the indirect effects of social support and psychological resilience on the association between perceived discrimination and subjective well-being, the hypothesized model was tested using structural equation modeling. This method fits the data using linear regression for subjective well-being, social support, and psychological resilience, controlling for demographic variables that were significantly associated with each outcome. Specifically, sex, situation of parents, and subjective family socioeconomic status were controlled for in the regression analysis of subjective well-being. Grade, parental educational level, and subjective family socioeconomic status were controlled for in the regression analysis of social support. Sex, grade, parental educational level, situation of parents, and subjective family socioeconomic status were controlled for in the regression analysis of psychological resilience. Multicollinearity was not violated, as all variance inflation factor values were less than 1.75. Discriminant validity was also confirmed, as the heterotrait-monotrait ratio of correlations (HTMT) values were significantly below the critical value of 0.9 (Henseler et al., 2015; Franke and Sarstedt, 2019). Maximum likelihood estimation was used. We used a Bootstrapping method (5000 bootstrap samples) to estimate standard errors and 95% bias-corrected confidence intervals (CIs) for the model effects (Shrout and Bolger, 2002). Consistent with general recommendations, model fit statistics, namely comparative fit index (CFI), Tucker–Lewis index (TLI), root mean square error of approximation (RMSEA), standardized root mean squared residual (SRMR), and chi-squared test (Jackson et al., 2009), were assessed. Both sexes were included in a single mediation model since the associations between perceived discrimination and social support, between social support and psychological resilience, between psychological resilience and subjective well-being, and between perceived discrimination and subjective well-being were not moderated by sex. This decision was further supported by the multi-group structural equation model, which indicated no statistically significant sex differences in direct, indirect, and total effects (bias-corrected bootstrap CIs for sex differences included zero; Table 2).

**TABLE 2:** Results from multi-group structural equation model.

Effect path	Boys		Girls		Sex differences	
	Estimate	95% boot CI	Estimate	95% boot CI	Estimate	95% boot CI
Total effect	−0.38***	[−0.48, −0.29]	−0.49***	[−0.58, −0.40]	−0.10	[−0.24, 0.03]
Direct effect: PD→SWB	−0.19***	[−0.28, −0.10]	−0.23***	[−0.32, −0.14]	−0.04	[−0.17, 0.09]
Total indirect effect	−0.20***	[−0.26, −0.14]	−0.26***	[−0.33, −0.20]	−0.07	[−0.15, 0.03]
Indirect effect 1: PD→SS→SWB	−0.07***	[−0.13, −0.04]	−0.08***	[−0.12, −0.05]	−0.01	[−0.06, 0.05]
Indirect effect 2: PD→PR→SWB	−0.08***	[−0.13, −0.04]	−0.14***	[−0.19, −0.10]	−0.06	[−0.12, 0.01]
Indirect effect 3: PD→SS→PR→SWB	−0.04***	[−0.07, −0.02]	−0.04***	[−0.06, −0.03]	−0.00	[−0.03, 0.03]
Model fit	χ^2^[26] = 24.83, *p* = 0.529, CFI = 1.00, TLI = 1.00, RMSEA = 0.00, 90% CI = [0.00, 0.04], SRMR = 0.02					

***p < 0.001; PD, perceived discrimination; SWB, subjective well-being; SS, social support; PR, psychological resilience.

## 3 Results

### 3.1 Common method bias test

Harman’s single factor test was used to test the common method bias, and exploratory factor analysis (EFA) was conducted for all the questions in the scale (Podsakoff et al., 2003). The results showed that there were nine factors with eigenvalues greater than 1, and the explanatory variation rate of the first factor was 25.02%, which was less than the critical value criterion of 40% (Podsakoff et al., 2003). Thus, common method bias was not a significant concern in this study.

### 3.2 Correlations among perceived discrimination, subjective well-being, social support, and psychological resilience

Pearson correlations were used to analyze correlations among perceived discrimination, social support, psychological resilience, and subjective well-being. The means, standard deviations, and correlations for each variable are presented in Table 3.

**TABLE 3:** Means, SDs and correlations.

Variables	Mean	S.D.	1	2	3	4
1 Perceived discrimination	13.13	5.61	1.00			
2 Social support	44.69	8.25	−0.38***	1.00		
3 Psychological resilience	52.53	10.27	−0.49***	0.54***	1.00	
4 Subjective well-being	18.89	5.66	−0.48***	0.53***	0.61***	1.00

***p < 0.001.

Perceived discrimination is significantly and negatively related to subjective well-being, social support, and psychological resilience (all *ps* < 0.001). Social support and resilience are positively correlated with subjective well-being (all *ps* < 0.001). We also find a significantly positive relationship between social support and resilience (*p* < 0.001).

### 3.3 Mediation of social support and psychological resilience

**FIGURE 1:**
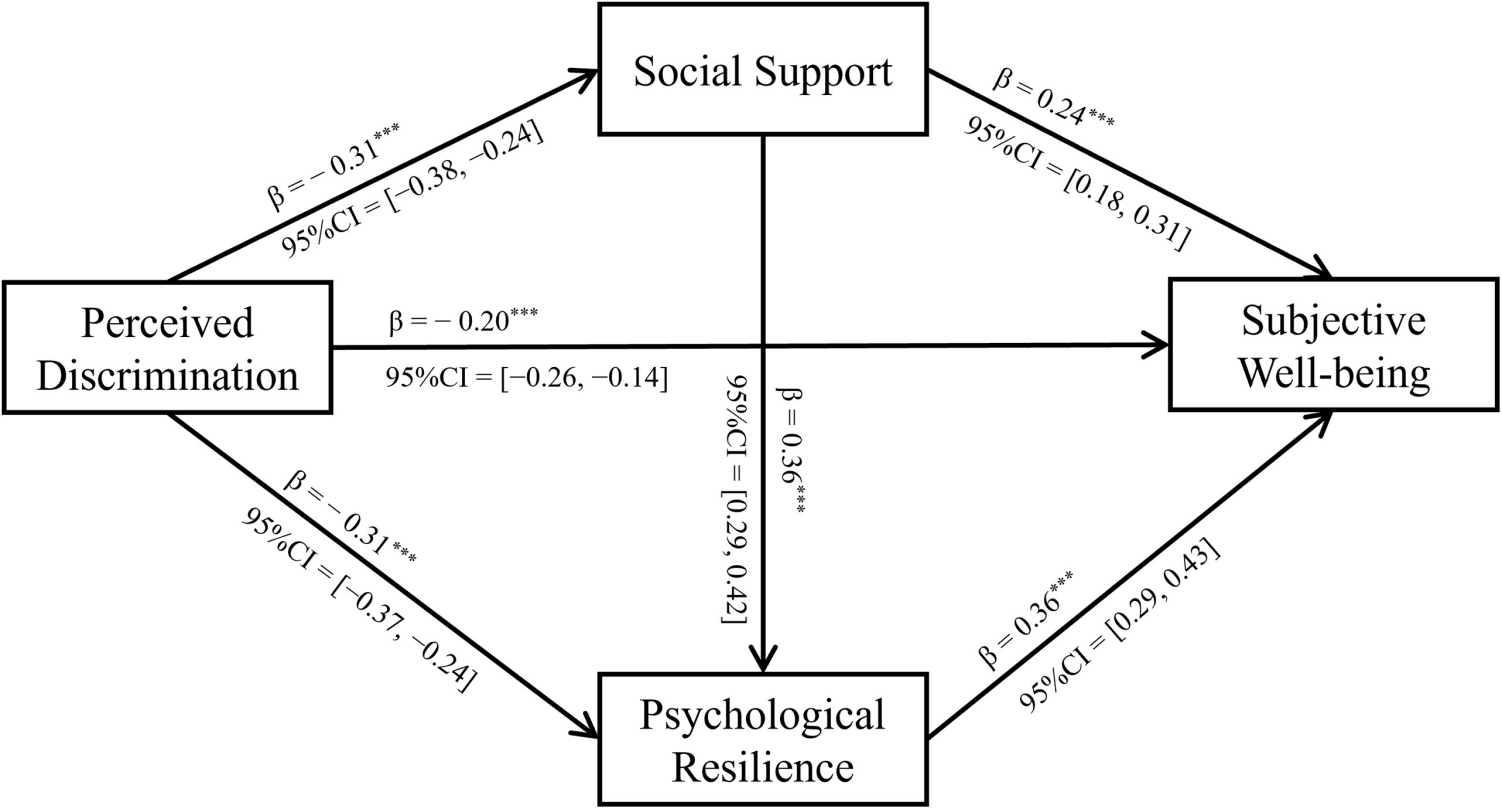
Mediation analysis: social support and psychological resilience mediating the relationship between perceived discrimination and subjective well-being. ***p < 0.001.

The standardized path coefficients were shown in Figure 1. The model showed good fit to the data (χ^2^[14] = 18.08, *p* = 0.203, CFI = 0.99, TLI = 0.98, RMSEA = 0.02, 90% CI = [0.00, 0.04], SRMR = 0.02).

#### 3.3.1 Total and direct effects

Controlling for demographic variables shown in the Section “2.4 Data analysis,” higher levels of perceived discrimination were associated with lower levels of subjective well-being, total effect (standardized regression coefficient) = −0.43, 95% CI = [−0.49, −0.36], *p* < 0.001. This association was weakened but remained significant when psychological resilience and social support were controlled for, direct effect = −0.20, 95% CI = [−0.26, −0.14], *p* < 0.001. This suggests that although psychological residence and social support may partially explain lower levels of subjective well-being associated with higher levels of perceived discrimination, perceived discrimination itself may still independently influence subjective well-being, or there may be additional unmeasured factors affecting the association between perceived discrimination and subjective well-being.

#### 3.3.2 Independent indirect effects

The lower levels of subjective well-being associated with higher levels of perceived discrimination were significantly mediated through social support (indirect effect = −0.08, 95% CI = [−0.11, −0.05], *p* < 0.001) and resilience (indirect effect = −0.11, 95% CI = −0.14, −0.08], *p* < 0.001). The degree of mediation by social support and resilience was around 18.60% and 25.58%, respectively. This suggests that higher perceived discrimination among LBC was linked to lower subjective well-being through two distinct pathways: one through lower social support, and the other through reduced psychological resilience.

#### 3.3.3 Chain indirect effect

The lower levels of subjective well-being associated with higher levels of perceived discrimination was also partially explained by a path involving lower levels of social support leading to reduced psychological resilience, the chain indirect effect of social support and psychological resilience = −0.04, 95% CI = [−0.06, −0.03], *p* < 0.001. The chain indirect effect of social support and psychological resilience explained around 9.30% of the association between perceived discrimination and subjective well-being. This suggests that higher levels of perceived discrimination were linked to lower social support, which in turn was associated with reduced psychological resilience, ultimately resulting in lower levels of subjective well-being.

### 3.4 Potential sex differences in the mediation analysis

A multi-group structural equation model was further conducted to test whether the total, indirect, and direct effects were the same across sex. The demographic variables controlled for, as well as the method used to calculate the effects and standard errors, were the same as those in the mediation analysis. The results indicated that sex did not moderate the indirect effects of social support and psychological resilience on the association between perceived discrimination and subjective well-being, as well as the direct and total effects of perceived discrimination on subjective well-being, as the bias-corrected bootstrap CIs for sex differences included zero (Table 2).

## 4 Discussion

This study, applying the stress process theory to the context of LBC and discrimination, utilized structural equation modeling to examine the mechanisms linking perceived discrimination to subjective well-being among Chinese LBC. Findings showed that higher perceived discrimination was significantly associated with lower subjective well-being. This association was partially explained by social support and psychological resilience directly, as well as a path involving lower levels of social support leading to reduced psychological resilience. These findings have important implications for mental health education, discussed near the end of this concluding section.

Consistent with Hypothesis 1 and previous studies (Pascoe and Richman, 2009; Schmitt et al., 2014; Zhang et al., 2020; Hu et al., 2022), we found that higher perceived discrimination was significantly associated with lower levels of subjective well-being among LBC. According to the stress process theory, LBC are often exposed to both blatant and subtle forms of discrimination, such as verbal or physical harassment, being ignored, or excluded (Zhao et al., 2016, 2020), which functions as a chronic stressor associated with adverse mental health outcomes. The negative link between perceived discrimination and subjective well-being among LBC can be understood through mechanisms such as feelings of rejection (Leary et al., 1995) or negative internalization (Jost and Banaji, 1994). These discrimination experiences negatively impact individuals’ mental health by threatening their need for acceptance and inclusion (Wirth and Williams, 2009; Ma et al., 2022). Our results further support the notion that perceived discrimination poses a significant risk to the psychological well-being of LBC, highlighting the importance of addressing systemic stigma for this vulnerable population.

Consistent with the stress process model, which posits that exposure to stress can increase the risk of poorer mental health through the depletion of social resources, the current study found that the association between perceived discrimination and subjective well-being was partially explained by social support. This supports Hypothesis 2 and is consistent with previous studies (Prelow et al., 2006; Jia and Liu, 2017; Fu et al., 2024). This result also verified the social support deterioration deterrence model (Kaniasty and Norris, 1993; Norris and Kaniasty, 1996). One possible explanation is that perceived discrimination implies a rejection or exclusion of the targeted group (Schmitt et al., 2014). LBC, who perceive themselves as being discriminated against, may be inclined to avoid interpersonal interactions to protect themselves from social rejection (Zhang et al., 2020), which limits their access to potential sources of social support. In addition, perceptions of everyday injustice or negative feelings of rejection can reduce individuals’ help-seeking, preventing the establishment of social networks between youth and society (Cristini et al., 2011; Mao et al., 2022). When individuals perceive inadequate social support (perceived unwillingness to help, listen, etc.), various mental health problems (anxiety, depression, burnout, etc.) may arise (Guilaran et al., 2018; Xiang et al., 2020; Chang et al., 2022).

Psychological resilience also partially explained the association between perceived discrimination and subjective well-being in LBC, providing support for Hypothesis 3 and aligning with the stress process model’s view of personal resources as mediators. This finding also aligns with previous research (Han and Long, 2020; Lv et al., 2021). According to symbolic interaction theory, LBC who are exposed to negative social stereotypes and prejudices–such as being perceived as unloved, unwanted, or problematic–may internalize these external evaluations. Discrimination is not just an external event but can be internalized (Fernández et al., 2015), damaging LBC’s self-concept and eroding the internal coping capacities (e.g., resilience) needed to manage adversity (Lv et al., 2021). Although psychological resilience is a protective factor against poorer mental health (Fritz et al., 2018; Ungar and Theron, 2020), perceived discrimination, as a chronic stressor, is linked to sustained stress, which may in turn be associated with reduced psychological resilience (Vargas et al., 2021), thereby resulting in lower subjective well-being (Zhou and Chen, 2024).

The results of the current study show the serial mediation effect of social support and psychological resilience between perceived discrimination and subjective well-being among LBC, confirming Hypothesis 4. These findings are consistent with recent studies supporting a serial mediation model in which social support and psychological resilience jointly influence mental health outcomes (Yan et al., 2019; Yuan et al., 2024). These findings also align with the resilience framework (Kumpfer, 2002), illustrating how perceived discrimination–acting as a chronic stressor–undermines both external support systems and internal psychological resilience among LBC, ultimately impairing their capacity to maintain well-being. Critically, these findings provide empirical support for the stress erosion hypothesis (Pearlin, 1989; Wheaton, 1994). The relationship between stress and psychosocial resources is complex. While the well-known stress buffering hypothesis posits that resources can mitigate the negative influences of stress, the stress erosion hypothesis argues that persistent and chronic stressors can overwhelm these resources. Our model demonstrates this erosion process in action: perceived discrimination as a chronic stressor erodes the LBC’s external support system (social support), which in turn leaves them without the necessary relational resources to build or maintain their internal coping capacities (psychological resilience), ultimately leading to the lower levels of subjective well-being.

Furthermore, the multi-group structural equation model revealed that sex did not moderate the indirect effects of social support and psychological resilience on the association between perceived discrimination and subjective well-being, nor the direct and total effects of perceived discrimination on subjective well-being, supporting Hypothesis 5. This finding suggests that the mechanism of stress erosion–whereby perceived discrimination depletes social support, which in turn erodes resilience–functions similarly for both left-behind boys and girls in our sample. This result suggests that the constant and widespread discrimination experienced by LBC may universally deplete their resources of social support and resilience, affecting their psychosocial well-being in a comparable manner regardless of sex.

While the context of Chinese LBC is unique–rooted in internal rural-to-urban migration and the hukou system–the mechanisms identified in this study are consistent with findings from other vulnerable populations. Research among immigrant children and ethnic minority youth has documented that both social support (Oppedal, 2011; Katsiaficas et al., 2013; Fernández et al., 2015), and psychological resilience (Vargas et al., 2021; Cénat et al., 2022) partially explain the relationship between discrimination and health outcomes. Furthermore, a serial mediation pathway involving social support and resilience has also been identified in people living with HIV (Yuan et al., 2024). This suggests that the stress erosion process, wherein chronic discrimination depletes external and internal resources, may represent a pathway impacting the mental health of stigmatized populations across different cultures.

Our findings have several practical implications. Perceived discrimination was negatively associated with subjective well-being among LBC. Therefore, to improve their well-being, eliminating discrimination and prejudice against LBC is the first step. In cases where discrimination cannot be completely eliminated, teachers and caregivers should pay more attention to LBC’s experiences of discrimination and guide them to formulate correct attributions regarding perceptions of discrimination. This would help eliminate the negative emotional experiences caused by perceived discrimination (Ma et al., 2022). Meanwhile, interventions aimed at improving self-acceptance (Fernández et al., 2015) and enhancing a sense of control (Szalacha et al., 2003) can help prevent the internalization of prejudice and discrimination. Our findings highlight the important roles of social support and psychological resilience in the relationship between perceived discrimination and subjective well-being. Thus, tailored psychosocial interventions that aim to enhance LBC’s social network and improve their resilience could facilitate the mitigation of the detrimental effects of discrimination on mental health. To build psychological resilience, group psychological counseling focusing on emotional regulation and coping skills can be utilized to enhance the psychological resilience of LBC in combating internalized discrimination (Ma, 2021). Resilience-based intervention programs (e.g., setting positive expectations, improving self-efficacy), which are incorporated into regular school hours, have also shown positive outcomes (Qu et al., 2024). Meanwhile, effective programs enhancing social support are warranted. Educating LBC about the importance of social support and the availability of associations that can provide it is a key element in an intervention program (Fernández et al., 2015). Promoting regular communication between LBC and their parents through digital platforms (Xie et al., 2024), as well as organizing activities to enhance social bonding at the level of family caregivers, school teachers, peers, and the community (Su et al., 2017), can help LBC build a strong social support network. Crucially, our serial mediation finding implies that these interventions must be integrated. As our stress erosion model shows, strengthening social support may be a prerequisite for building resilience. Interventions that only teach coping skills (internal) to a child who remains socially isolated (external) will likely fail. We advocate for integrated programs that, for example, use group therapy to simultaneously build coping skills and foster a new peer-support network, addressing both mediators in our model.

This study also has some potential limitations. First, although the instruments showed good reliability and validity, the current study used a single evaluation method, and all the data were based on self-reports of LBC. The use of paper-and-pencil surveys with verbal explanations for younger pupils, while intended to improve comprehension, may have introduced interviewer effects or social desirability bias. Future studies should use multi-source data (e.g., peer or teacher reports). Second, our study design was cross-sectional, which cannot establish a causal relationship; longitudinal or experimental studies should be performed to verify our results. Future longitudinal research with at least three time points is crucial to pinpoint when the erosion of social support begins and subsequently triggers a decline in psychological resilience within this population. Third, apart from demographic characteristics, we were unable to account for other unmeasured confounders that may have influenced the associations among perceived discrimination, social support, psychological resilience, and subjective well-being, such as duration of parental absence and peer attachment. Finally, the sample size and limited geographical scope (i.e., only a single county in southwest China) limit the extent to which the findings of this study can be generalized. Thus, there is still some uncertainty about whether our findings can be generalized to all LBC in Sichuan Province or elsewhere in the country.

## 5 Conclusion

The current study adds further support to previous findings regarding the negative impact of perceived discrimination on individuals and delineates the path of this impact through a combination of social and individual factors. The findings highlight the importance of considering social support and resilience in the effect of perceived discrimination on subjective well-being among LBC. Reducing discrimination, enhancing psychological resilience, and providing increased social support are effective measures for intervening in the mental health of LBC.

## Data Availability

The raw data supporting the conclusions of this article will be made available by the authors, without undue reservation.
